# Feeding Infants with Asses’ Milk

**Published:** 1889-04

**Authors:** 


### FEEDING INFANTS WITH ASSES' MILK.

[From the *Scientific American*, March 16, 1889.]

In the Hospital for Infants' Diseases, situated in Sabres street, there exists a section for rickety boys and girls, whose miserable aspect produces an impression of pain upon the mind—unfortunate beings who have inherited the organic vices of their parents, and who suffer from anemia's cruel tortures.

The administration of the hospital is arranged in two separated pavilions, where there is much ventilation, with large windows that look out upon a garden, and whose walls have double rows of willow cradles perfectly equipped. The newly born receive here the personal care of the establishment, beginning with being weighed in the balance the same day they make their appearance, the operation being frequently repeated almost every month, in order to determine with exactness the development of the child. The little one is subjected to an especially nutritious diet of the most tonic kind, if it had been previously fed from a refractory goat liable to convey contagious germs, it having been found by experiment that the milk of this animal, although possessing nutritive principles of the most salutary kind, presents the inconvenience of communicating by absorption the effects of those nervous accidents to which the goat is subject.

The public charities of Paris, advised by the wise doctors of medicine, have substituted for the milk of goats that of the ass, and have installed an ample yard near the pavilion of the rickety and scrofulous children, which is only separated by a short covered passageway.

The nurses, dressed in dark gowns with white caps and aprons, each carrying a child on the right arm and a little seat in the left hand, present themselves in exact turn to the women who have charge of the animals, and they hold the child, applying its lips to the teats of the docile animal. The children suck with avidity the liquid nutriment, which is fresh and of agreeable taste.

The Administration of Public Assistance of Paris has calculated that one young ass is able to lactate abundantly for a space of nine or ten months, and when this period has passed they are sold and replaced by others. It is well known that the milk of asses, by its vivifying qualities and its nutritious principles, assimilates in a great degree the milk of the nurse, and these disinherited and sick children, enjoying its beneficial effects by its permanent and methodical use, are restored little by little to health and vigor.—*La Illustracion Espanola.*

